# Speech-in-Noise Ability and Signal to Noise Ratio Predict the Timing of Hearing-Impaired Listeners’ Intertalker Saccades When Observing Conversational Turn-Taking: An Explorative Investigation

**DOI:** 10.1097/AUD.0000000000001701

**Published:** 2025-07-15

**Authors:** Martha M. Shiell, Sergi Rotger-Griful, Martin A. Skoglund, Gitte Keidser, Johannes Zaar

**Affiliations:** 1 Eriksholm Research Centre, Oticon A/S, Snekkersten, Denmark; 2 Division of Automatic Control, Department of Electrical Engineering, The Institute of Technology, Linköping University, Linköping, Sweden; 3 Department of Behavioural Sciences and Learning, Linneaus Center HEAD, Linköping University, Linköping, Sweden; 4 Hearing Systems Section, Department of Health Technology, Technical University of Denmark, Kongens Lyngby, Denmark.

**Keywords:** Communication, Conversation perception, Eyetracking, Hearing-impaired, Speech-in-noise

## Abstract

**Objectives:**

We explored the hypothesis that, when listeners visually follow the turn-taking of talkers engaged in a conversation, the timing of their eye movements is related to their ability to follow the conversation.

**Design:**

We made use of a re-purposed dataset where adults with hearing impairment (N = 17), assisted by hearing aids, observed audiovisual recordings of dyadic conversations presented via a television screen and loudspeakers. The recordings were presented with multitalker babble noise at four signal to noise ratios (SNRs), in 4-dB steps ranging from –4 to 8 dB, to modulate the participants’ ability to follow the conversation. We extracted time windows around conversation floor transfers (FTs) in the stimulus where participants reacted by moving their gaze from one talker to the next, termed FT-intertalker saccades (ITS). We recorded the timing of this eye movement relative to the onset of the new talker’s speech. In addition, participants completed a separate word-recognition test to measure their speech perception in noise (SPIN) ability at the same SNRs as used for the conversation stimuli. We predicted that the timing of FT-ITS would be delayed with difficult SNR levels and for listeners with low SPIN ability. The effect of SPIN ability was tested first as a continuous variable, and subsequently with participants divided into high and low SPIN-ability groups.

**Results:**

Multilevel linear modeling showed that the timing of FT-ITS was predicted by SNR condition and SPIN group, but no effect was found for SPIN ability as a continuous variable. Post hoc comparisons (uncorrected for multiple comparisons) indicated that delayed FT-ITS were associated with low SPIN ability, and both the hardest and easiest SNR conditions. The full model accounted for 34.5% of the variance in the data, but the fixed effects of SPIN and SNR together accounted for only 2.3%.

**Conclusions:**

Although the results should be interpreted with caution due to limitations in the experiment design, they provide preliminary support that FT-ITS timing can be used as a measure of hearing-impaired listeners’ ability to follow a conversation. This first exploration of this question can serve future studies on this topic, providing guidance on the range of perceptual difficulty where this measure may be sensitive, and recommending a modeling approach that takes into account differences between stimuli.

## INTRODUCTION

Coordination of turn-taking between talkers is an important feature of effective communication. Particularly, the turn-taking must occur in a timely manner that avoids overlap between talkers or long pauses. To achieve this, it is essential that conversational partners are able to predict the end of their counterpart’s speaking turns (Barthel & Sauppe 2019). Syntactic and lexical information from the talker’s speech, and cues from the talker’s pitch, level, and speaking rate, contribute to this timing prediction (Gravano & Hirschberg 2011). Visual cues from the talker are also informative, but audio information is essential for a precisely timed prediction (Latif et al. 2017). Unsurprisingly then, for people with hearing impairment, degraded auditory processing is thought to impair turn-taking perception, such that this population shows delayed and more variable turn-taking than that of normal-hearing (NH) interlocutors when participating in a dyadic conversation (Sørensen et al. 2020). These deficits in turn-taking are theorized to contribute to the challenges that hearing-impaired (HI) people face in social communication contexts (Sørensen 2021). As such, turn-taking perception may indicate some part of a listener’s ability to function in real-world communication and thus merits investigation as a potential ecologically valid measure of hearing impairment.

Numerous studies have demonstrated that, when attending a prerecorded audiovisual conversation, listeners will tend to move their eyes from one talker to the other (an intertalker saccade [ITS]) when the talkers transition between their speaking turns (a conversation floor transfer [FT]) (Tice & Henetz 2011; Casillas & Frank 2012; Foulsham & Sanderson 2013; Hirvenkari et al. 2013; Keitel et al. 2013; Dawson & Foulsham 2022; Hadley & Culling 2022; Hidalgo et al. 2022; Shiell et al. 2023). This behavior, termed FT-ITS (floor-transfer intertalker-saccades) is the focus of the current investigation. Although our study is limited to the study of passive viewing of a conversation, the basic FT-ITS behavior occurs also when individuals participate in a live group interaction (Holler & Kendrick 2015; Dawson & Foulsham 2022; Hadley & Culling 2022).

The timing of FT-ITS indicates that listeners often anticipate the end of a talker’s turn (e.g., Casillas & Frank 2012; for a summary of evidence supporting this, see the discussion of Shiell et al. 2023). This anticipation is notable because it requires that a listener simultaneously processes incoming sensory information to monitor for turn-taking cues, makes predictions about future incoming information, and uses these predictions to plan and execute a precisely timed eye movement. In theory, the coordination of these simultaneous processes can be studied by examining the timing of FT-ITS. In line with this, some evidence indicates that the timing of FT-ITS is sensitive to the clarity of turn-taking cues: When cues are more explicit, such as in question-and-answer conversation exchanges, FT-ITS occur earlier than when cues are more ambiguous (Casillas & Frank 2012). Inversely, when turn-taking cues are obscured, for example, by removing the sound of an audiovisual conversation (Hirvenkari et al. 2013) or degrading it by means of vocoding (Hidalgo et al. 2022), FT-ITS are delayed.

The current investigation is a preliminary test of the hypothesis that the timing between FT-ITS is related to the clarity of turn-taking cues, where poor perception of these cues leads to delayed FT-ITS. Building on previous work in NH listeners, we explored if this relationship holds in adults with hearing impairment. To the best of our knowledge, this is the first application of FT-ITS in this population, and can thus serve to guide future research. We made use of a previously collected, unpublished, eye-tracking dataset where HI adults with hearing aids observed a prerecorded, unscripted conversation between 2 talkers. Clips of the conversation were presented with multitalker babble noise at different signal to noise ratios (SNR) that modulated the level of difficulty for listeners. Note that the SNR conditions were not equally sampled, with particularly fewer measurements in the easiest SNR condition. In a separate word-recognition task, listeners’ speech-in-noise (SPIN) ability was measured. We used an exploratory multilevel modeling approach to test if the timing of FT-ITS was predicted by the SNR of the conversation and/or individual listeners’ SPIN ability, two factors that are related to the clarity of speech and therefore should affect the perception of turn-taking cues in the conversation. Building on the previous research discussed above, we predicted that low SNR and poor SPIN ability would impair turn-taking perception and hence delay FT-ITS.

## MATERIALS AND METHODS

### Participants

For the current investigation, we made use of data that were collected for a different project (N = 8) and a follow-up pilot study 1 yr later (N = 13). The research abided by established international research codes as per the declaration of Helsinki. Participants were recruited from an internal database and all gave informed written consent.

Twenty-one HI adults participated. After data collection, 1 participant was excluded due to technical malfunctions during the experiment, and 3 more were excluded because of insufficient data, with more than 40% of their eye-tracking data missing due to poor pupil visibility. The final sample consisted of 17 participants (average years old: 71, range years old: 61 to 86, 10 women and 7 men). Details on their hearing loss are presented in Table 1. Participants reported that they could see the stimuli clearly without the use of glasses or contact lenses.

**TABLE 1.** Participant information

SPIN Group	SPIN	PTA	Hearing Loss Asymmetry	SNR Conditions
Low				
1	51	55	15	−4, 0, 4
2	58	75.6	5	−4, 0, 4
3	65	57.5	15	0, 4, 8
4	69	46.2	5	−4, 0, 4
5	69	45.6	5	−4, 0, 4
6	71	68.8	15	−4, 0, 4
7	71	61.9	5	−4, 0, 4
8	73	63.8	10	−4, 0, 4
High				
1	75	36.9	25	−4, 0, 4
2	80	60	10	0, 4, 8
3	82	43.8	5	−4, 0, 4
4	82	31.3	10	0, 4, 8
5	85	46.9	15	−4, 0, 4
6	89	41.9	10	−4, 0, 4
7	90	45.6	10	0, 4, 8
8	90	30.6	10	−4, 0, 4
9	92	51.3	15	−4, 0, 4

SPIN group was determined by splitting participants according to their average scores, on a word-recognition task with 4- and 0-dB SNRs, with both audio-only and audiovisual presentations. PTA is calculated across left and right ears at 500, 1000, 2000, and 4000 Hz. Hearing loss asymmetry refers to the maximum difference between the left and right ears in at least three of four frequencies tested. SNR conditions refer to the conditions presented for the eye tracking and SPIN measurements.

PTA, pure-tone average; SNR, signal-to-noise ratio; SPIN, speech-in-noise.

### Materials

**Fig. 1.**
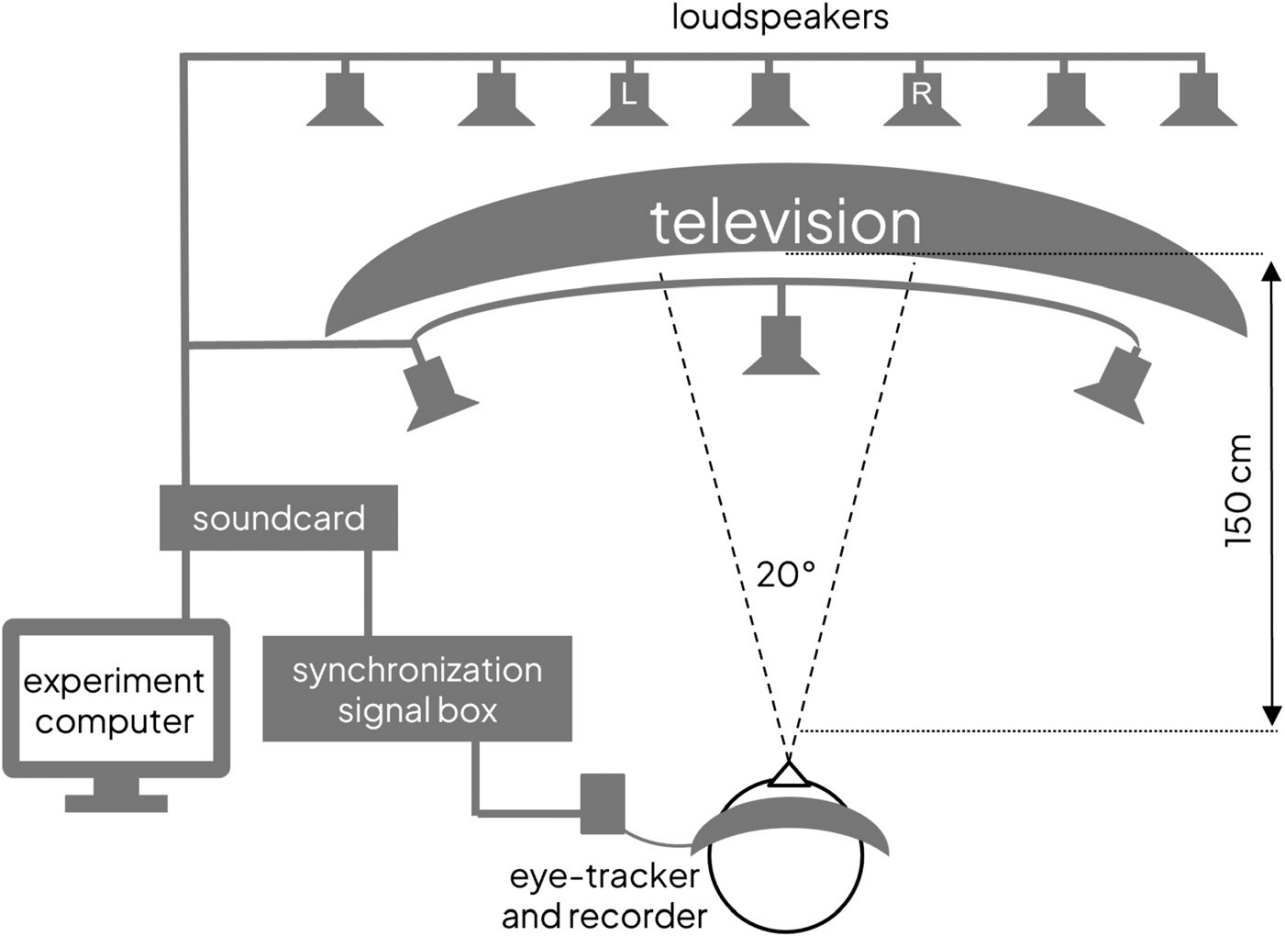
Schematic of the experiment setup, adapted from Skoglund et al. (2022). Two talkers were presented on the left and right sides of the television screen, and their speech was played from the loudspeakers labeled “L” and “R,” respectively. All other loudspeakers played the multitalker babble noise. The loudspeakers behind the television screen were directly above it and covered by an acoustically transparent cloth. The loudspeakers in front of the television screen were at floor level, visible to the participant but not obstructing the screen view.

The hardware and software for stimuli presentation and eye-tracking data collection were identical to those described in Shiell et al. (2023). This experiment setup is illustrated in Figure 1. The stimuli were presented visually on an 88” curved Samsung television screen and acoustically with 10 loudspeakers that were distributed above and below the screen. The loudspeakers were interfaced to the experiment computer via two linked soundcards (RME Fireface 400 and Hammerfall DSP Multiface II). Eye movements were measured with Tobii Pro 2 Eye tracking Glasses (Tobiipro, Sweden, https://www.tobiipro.com). Eye-tracking data collection was synchronized to stimulus presentation via a synchronization signal that was sent through the soundcard and a custom-made trigger box to the eye-tracker.

**Fig. 2.**
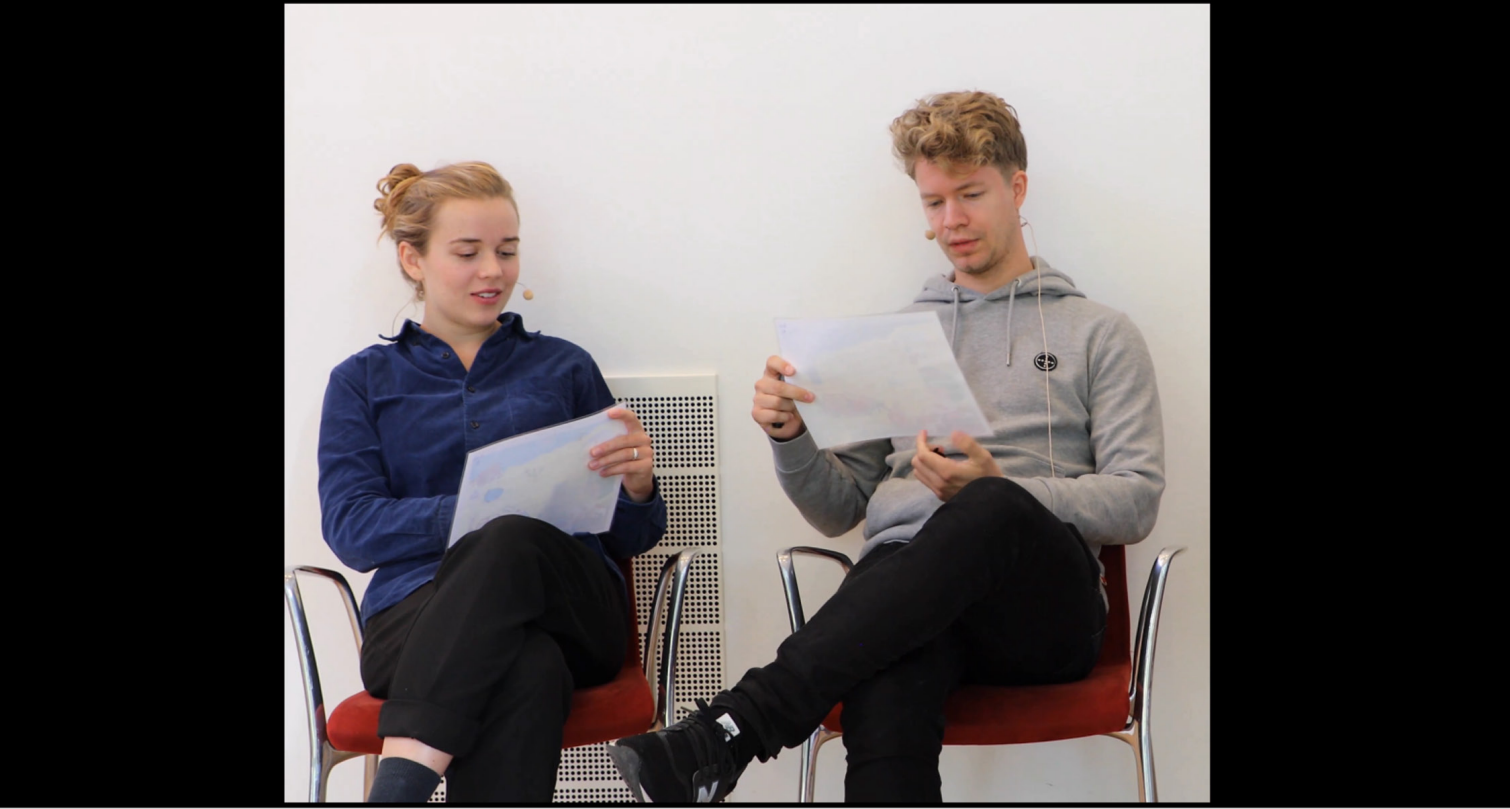
Screenshot from the video of an example conversation segment used as stimuli (adapted from Skoglund et al. 2022). Two of the 4 different talkers used in the experiment are shown. The video filled an 88” television screen, such that the size of the talkers on the screen was approximately life-like. The talkers’ bodies were constrained only by the position of the chairs, which remained constant throughout all of the trials used in the experiment.

For the eye-gaze measurement, the stimulus consisted of audiovisual recordings of 2 talkers (see Fig. 2 for an example) completing a spot-the-difference task (Baker & Hazan 2011), presented with 10-talker babble noise. This noise was chosen to provide relatively steady energetic masking that preserved realistic acoustic properties of speech, but without semantic information that might introduce informational masking. The multitalker babble signal was identical at each loudspeaker. Each talker in the conversation was presented at an average sound pressure level (SPL) of 62 dB. The level of the babble noise was varied to create four different SNR conditions: −4, 0, 4, and 8 dB. However, each participant completed only three of these conditions, as shown in Table 1. This occurred because the initial data collection used the −4-, 0-, and 4-dB SNR conditions only, which were chosen to capture behavior in response to a range of difficulty levels. In the follow-up data collection, the −4-dB condition was removed after we observed that it was frustrating for participants, and the 8-dB condition was added instead.

The conversation was split into segments that varied from 9 to 39 sec in duration, cut around the natural starting and stopping points in the speech. A total of 19.7 min of conversation was used over all segments. Due to the realistic nature of the conversation, the number of FTs varied in each conversation segment, with a median of 11 FTs per segment, and a range of 2 to 22 FTs.

The SPIN stimuli were monosyllabic Danish words spoken by an adult female (Dantale I corpus, Elberling et al. 1989). These were presented with a stationary background noise from a single loudspeaker centered in front of the participant. Matlab 2016 (Mathworks, USA, https://mathworks.com) controlled the stimulus presentation. The target speech was presented at 62-dB SPL and the noise level was varied to create the same SNR conditions that each participant was exposed to in the eye-tracking measurement (Table 1). Each SNR condition was presented in both an audio-only and an audiovisual condition. In the audio-only condition, the female talker’s voice was accompanied by a black TV screen. In the audiovisual condition, the female talker’s face was presented on the TV screen speaking the words. The talker was shown from the shoulders up and covered the majority of the screen.

Participants were aided by Oticon Opn S1 mRite hearing aids in both the eye-tracking and SPIN measurements to ensure stimulus audibility. For all participants except one, the hearing-aid model was the same as that which they habitually used. The hearing aids were programmed with a VAC+ fitting rationale that followed each participant’s prescription, but without noise reduction or directionality, and fitted with closed-style domes.

### Test Procedure

For both the eye-tracking and the SPIN measurement, participants were seated approximately 1.5 m from the television screen. They were verbally instructed on the tasks, given examples of the stimuli, and an opportunity to practice. The stimuli were presented in blocks and the order of these blocks was counterbalanced between participants.

The experiment began with the eye-tracking measurement. Participants were fitted with the eye-tracker glasses and presented with 45 trials split into 3 blocks of 15 trials each. Each trial started with a white fixation cross on a black screen accompanied by the background multitalker babble noise for 3 sec, followed by a conversation segment. The trial ended with the presentation of a three-option multiple-choice question on the screen that asked about the content of the conversation, and the participant made a response by button-press on a number keypad. The question was meant to motivate participants to attend to the conversation. All participants responded to every question with a median of 67% correct (interquartile range: 27%). This low score may have been driven by difficulty understanding the speech at the low SNR levels, and by those participants with more severe hearing loss. However, as the questions were not standardized in terms of difficulty and not equally presented across the SNR conditions, the participants’ scores will not be discussed further here.

Each block included five trials from each of three SNR conditions, with the presentation of the SNR conditions ordered pseudo-randomly within the block, such that each SNR condition was not presented more than twice sequentially. This design, with the distribution of SNR conditions over the course of the experiment, was chosen to avoid concentrating the difficult trials in time for the comfort of the participants. The SNR condition associated with each conversation segment was counterbalanced between participants, such that the same conversation segment was used for different SNR conditions across participants. Participants were given the opportunity to take a break between blocks and the eye-tracker was recalibrated before starting the next block.

Following the eye-tracking measurement, we measured the participants’ SPIN. Six lists of 25 words each were used, with 1 list for each SNR condition in each of the audio-only and audiovisual conditions. Table 1 shows the SNR levels presented to each participant. Note that the same SNR levels were used in both eye-tracking and SPIN measurements. Participants repeated each word they heard after the word was presented. Responses were either scored live during the session or they were recorded with a video camera for later scoring. Scoring was completed by an experienced Danish audiologist. Our investigation was not concerned with differences between audio-only and audiovisual SPIN, and we expected that both would be relevant for listeners’ abilities to follow turn-taking cues. Therefore, to increase the reliability of the SPIN measure at the individual level, we averaged each participant’s performance across SPIN conditions to generate a single multimodal score. For this, we used only the 0- and 4-dB SNR conditions, because only these were completed by all participants. This average resulted in a score that was based on 100 words. Note that since scores on the audiovisual and audio-only conditions were correlated (for 0-dB SNR, *r* = 0.69, *p* = 0.0008; for 4-dB SNR, *r* = 0.62, *p* = 0.0035), this averaging also helped us avoid multicollinearity in our subsequent modeling analysis.

### Analysis

**Fig. 3.**
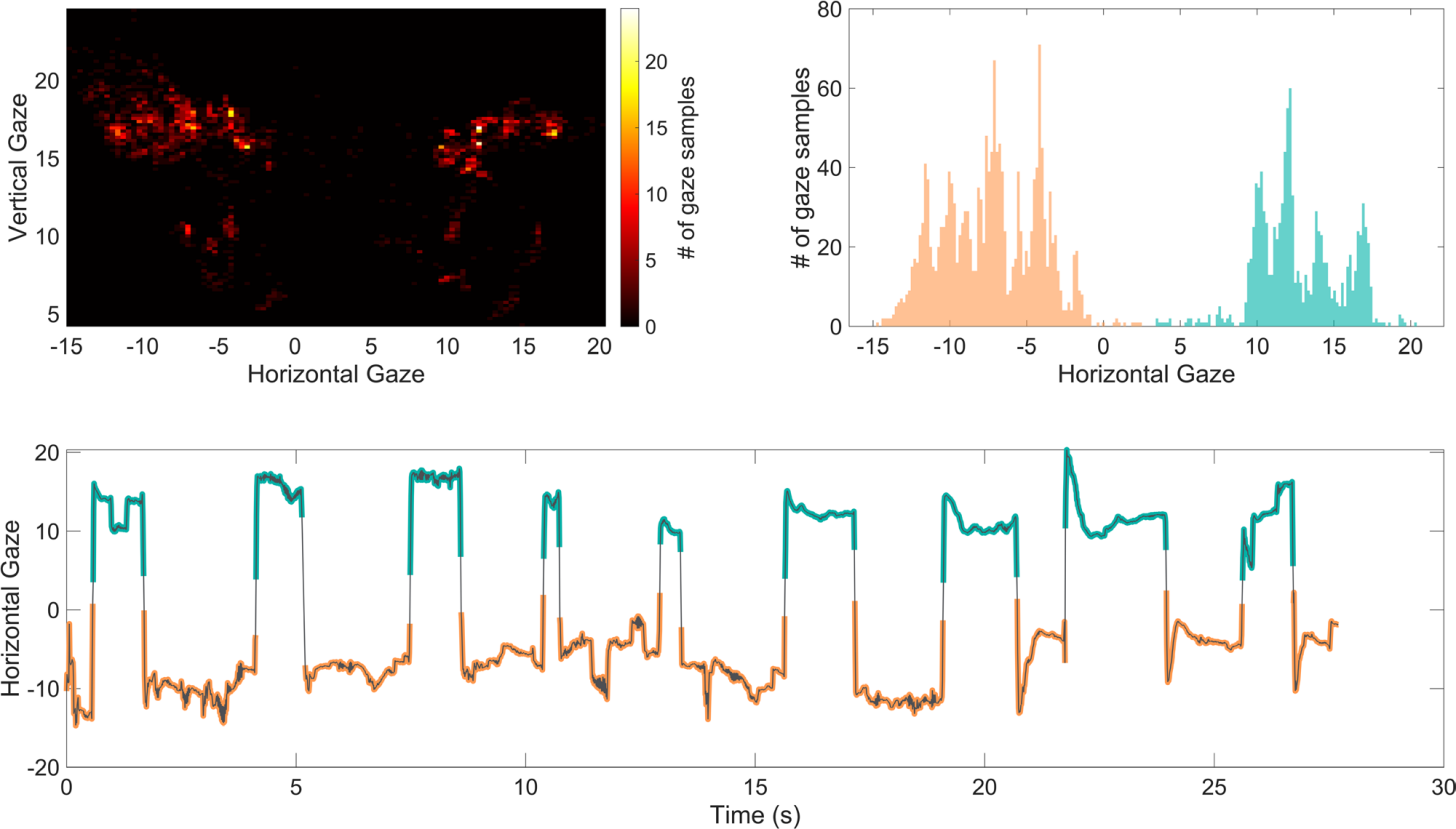
Example gaze data and automatic labeling from a single trial. In all panels, gaze position is represented in degrees of visual angle relative to the listener’s head. Listeners’ heads were not fixed during the eye-tracking measurement, and the absolute position of the gaze relative to the stimulus was not recorded. Top left: Distribution of gaze position, showing that the listener tended to direct their gaze toward two regions on the left and right of the scene. The spacing of these regions corresponds roughly to the position of the faces of the 2 talkers in the conversation stimuli (Fig. 2), which were approximately 20° apart from the viewing position of the listener (Fig. 1). Top right: Distribution of horizontal gaze position, labeled as belonging to one of two clusters, shown in orange and green, via a *k*-means clustering algorithm (*k* = 2). Bottom: Horizontal gaze position over time (gray line) and corresponding labels (orange and green). Intertalker saccades were defined as instances where the label changed.

Eye-tracking data were processed with TobiiPyGlassesSuite (De Tommaso & Wykowska 2019) and custom scripts in Matlab 2019a and R (version 3.6.1, https://www.r-project.org). Twenty-one trials (3% of the 765 trials collected) were excluded because they had more than 40% of their data missing. The eye-gaze data processing was identical to that of Shiell et al. (2023). The analysis procedure is based on the fact that listeners will tend to look at faces compared with other elements of a scene (Buswell 1935). In our experiment setup, the two talkers’ faces were positioned approximately 20° apart from one another, and thus produced a gaze pattern where most samples fell in two clusters over the expected face regions (see Fig. 3 for an example). These clusters were labeled automatically via a *k*-means algorithm (with *k* = 2) applied to the horizontal azimuth data from the eye with the least missing data. This was done after excluding extreme gaze positions (±25° around median gaze azimuth) that could bias the clustering. With this labeling, we observed that over all participants and trials, gaze was assigned to the cluster that was associated with the active talker 59.6% of the time.

ITS were then identified as changes in label that endured for at least 180 msec, which we estimated to be the minimum time for gaze to remain on one side of the midline between the two faces if it represented a true ITS. This included 40 msec for the eyes to travel from the midline to a talker’s face (approximately 10°), 100 msec for a minimum fixation time on that talker’s face, and 40 msec to return to the midline and change labels. It is likely that with this method, some saccades were mislabeled as ITSs when they were rather saccades towards other elements of the scene. We considered these as noise. The automatic labeling and ITS identification were visually checked for every trial. In 29 trials (4% of the 744 trials that had sufficient data), the labeling was subjectively deemed to be poor, but these trials were nonetheless left in the analysis to maintain an objective and automatic procedure.

Although eye-tracking data were collected for the full duration of each trial, only the data associated with FT events in the conversation stimulus were analyzed. To identify FT events, the acoustic conversation stimulus in each trial was analyzed using the same procedure as in Shiell et al. (2023). FTs were identified automatically and were defined as instances where one talker’s voice activity stopped and the other’s began, indicating the end of one’s turn and the start of the other’s. We excluded FTs if the two talkers overlapped in their speech, given that previous research has shown that listeners are likely to continue gazing at the previous talker in these cases (Shiell et al. 2023). With these criteria, across all of the conversation stimuli, there were 321 conversation FTs, with a median silent gap between the talkers of 280 msec and a range from 0 to 1.82 sec. From these, we excluded cases where we intuited that listeners’ may not have had sufficient time to make a FT-ITS due to a short duration of either the new talker’s turn or the silent gap between the two talkers’ turns. For this, we used minimum duration criteria of 400 and 100 msec, respectively, which excluded approximately 20% of the shortest silent gaps and turns. Note that this decision to control elements of the FT is consistent with what has been done in previous research. For example, Hidalgo et al. (2022) used scripted conversation stimuli and artificially set each FT silent gap to 500 msec.

Our criteria produced 253 FT events in the stimuli, which had a median silent gap of 280 msec (interquartile range: 281 msec). For each FT event, we selected a time window-of-interest that included the silent period before the onset of the new talker’s speech (to a maximum of 400 msec) and the start of the new talker’s turn (to a maximum of 1.4 sec). These timing cutoffs were based on observations from our earlier study (Shiell et al. 2023). For each FT, we limited the time window to exclude overlap with the end of the previous talker’s turn or any time period after the end of the new talker’s turn.

From the eye-tracking data associated with these time window-of-interest, we limited our analysis to horizontal gaze angle data in which the participant made a single ITS from the previous talker to the new talker, which occurred 1575 times (in 38% of the total occurrences). These 1575 occurrences broke down to 332, 527, 571, and 145 FT-ITS in each of the −4-, 0-, 4-, and 8-dB SNR conditions, respectively. Excluded were data where participants either made 2 or 3 ITS in the time window-of-interest (375 and 68 occurrences, respectively), no ITS (1800 occurrences), or a single ITS in the opposite direction (from new talker toward previous talker, 341 occurrences). Note that such variation in gaze behavior during conversation FTs has been observed in a previous study with NH participants engaged in a triadic conversation (Holler & Kendrick 2015), where it was reported that only 45% of conversation FTs elicited ITS toward the new talker.

For the selected FT-ITS, their timing was calculated relative to the onset of the new talker’s speech in each conversation FT, where negative values indicate that the FT-ITS occurred before the new talker began speaking. As per our analysis procedure (outlined earlier), this timestamp represents the point at which the gaze changed between the automatically labeled clusters. The median timing in our dataset was 260 msec, with an interquartile range from 60 to 460 msec.

We used the lmerTest package (Bates et al. 2015; Kuznetsova et al. 2017) to implement multilevel linear modeling to test our hypotheses. We tested whether we could predict the timing of the FT-ITS in each measurement based on two fixed effects: the SNR condition of the measurement, and the SPIN ability of the participant. Initially, we included SPIN ability by logit-transforming the participants’ word recognition scores. As can be seen in the Results, this implementation failed to improve the model. As an exploratory follow-up, we decided post hoc to test a simplified implementation of the SPIN measure where participants were divided into high and low SPIN groups, shown in Table 1, that were separated by the group’s median word-recognition score (Iacobucci et al. 2015). To mitigate the risk of a false positive with this implementation, we tested our original model for collinearity using the variance inflation factor (Zuur et al. 2010). Both of the fixed effects terms for SPIN and SNR had variance inflation factor = 1.0, indicating low collinearity between them.

Our initial model also included the interaction between SNR and SPIN, and two random effects: an intercept for each unique FT in the stimuli, and for each individual participant. These random effects terms allowed the model to fit the data independently for each unique FT shown in the experiment, and for each individual participant. This controls for the variance that is driven by either differences between FTs in the stimuli or differences between participants, making the model more sensitive to finding effects from the predictor-of-interest (i.e., SNR and SPIN ability).

The optimal model was determined by forward additive modeling and backward stepwise elimination with the Akaike Information Criterion (AIC), applying a significance criterion of *p* < 0.1. We subsequently ran *t* tests, uncorrected for multiple comparisons, on the estimated marginal means for each level of our conditions to describe the relationship between the four levels of SNR and the relationship between the SPIN groups. Finally, we ran the optimal model excluding each of the random-effect variables to describe how these factors contributed to the total variance explained.

## RESULTS

**Fig. 4.**
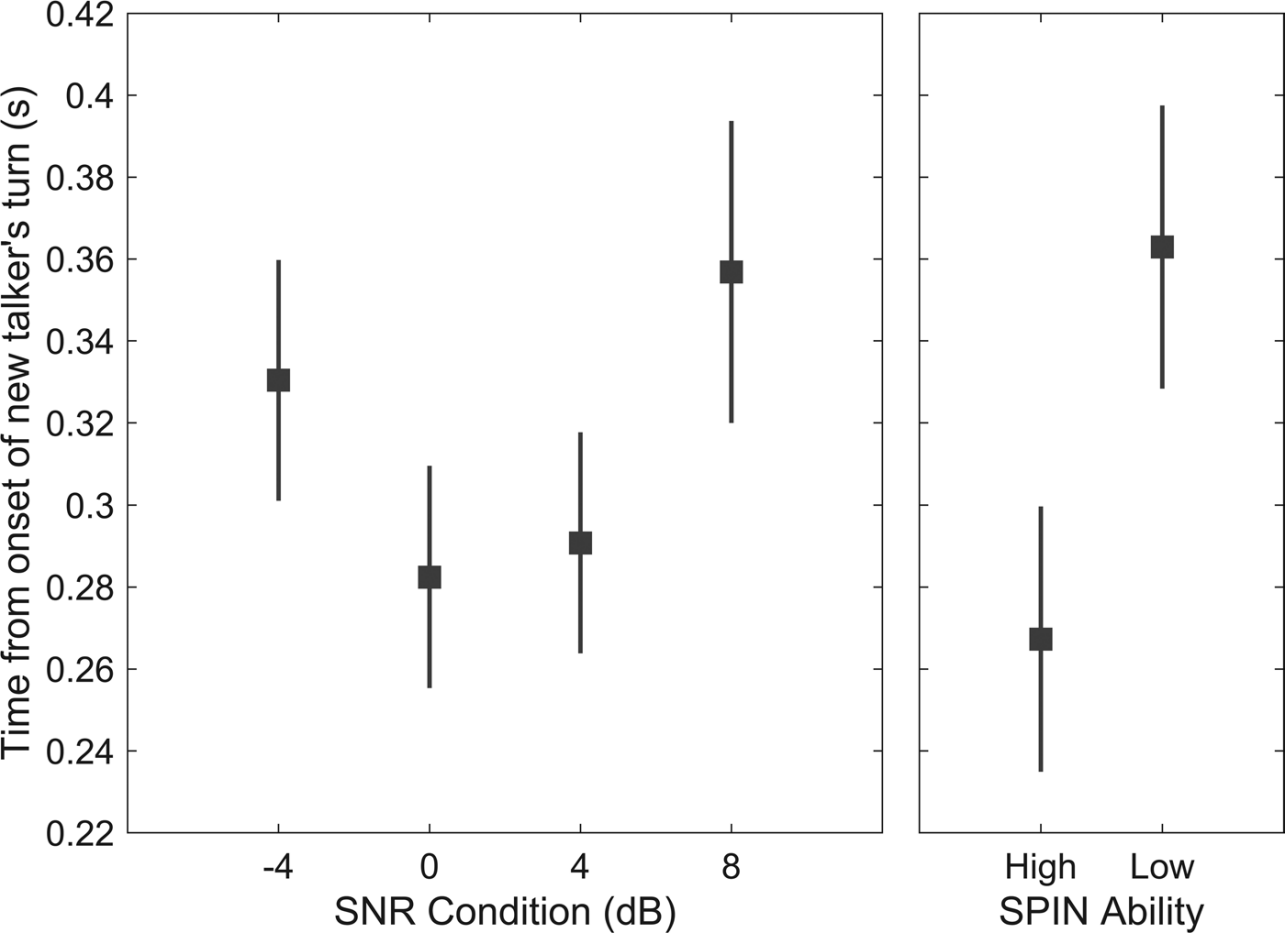
FT-ITS timing predicted by SNR condition and SPIN group. Estimated marginal means and SE bars for the fixed effects of SNR condition (−4, 0, 4, or 8 dB, estimated across SPIN groups, left panel) and SPIN group (high or low, right panel) from the optimal model: FT-ITS timing ~ SNR condition + SPIN group + (1|participant) + (1|FT instance). FT-ITS indicates floor-transfer intertalker-saccade; SNR, signal to noise ratio; SPIN, speech-in-noise.

**Fig. 5.**
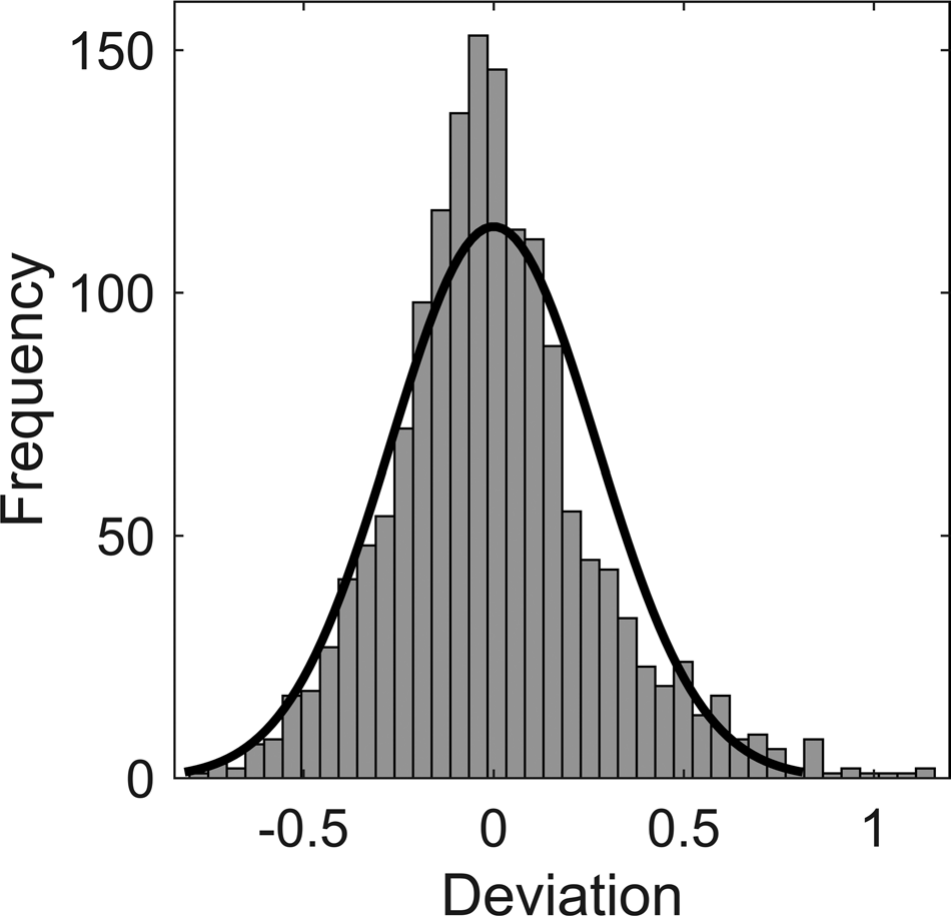
Model residuals overlaid with normal distribution. Residual variance from the optimal model: FT-ITS timing ~ SNR condition + SPIN group + (1|participant) + (1|FT instance). The black line shows the fit of a normal distribution. FT-ITS indicates floor-transfer intertalker-saccade; SNR, signal-to-noise ratio; SPIN, speech-in-noise.

FT-ITS timing was predicted by a combination of the fixed effects SNR [*F*_(3,1363)_ = 3.56, *p* = 0.014], SPIN group [*F*_(1,14)_ = 4.83, *p* = 0.045], and random intercepts for each unique FT and participant. The inclusion of a fixed-effects term for the interaction between SNR and SPIN group did not improve the model’s predictiveness [*F*_(3,1383)_ = 1.21, *p* = 0.30], nor did the use of the participants’ logit-transformed word-recognition scores as a measure of their SPIN ability [*F*_(1,15)_ = 2.74, *p* = 0.12], rather than grouping the participants into high and low SPIN ability. The estimated marginal means for each SNR level and SPIN group are shown in Figure 4 for the optimal model: FT-ITS timing ~ SNR condition + SPIN group + (1|participant) + (1|unique FT). This model generated a near-normal distribution of the residuals, shown in Figure 5. It explained 34.5% of the variance in FT-ITS timing, but only 2.3% of this variance was attributed to the fixed effects alone. Excluding the random effects parameter for each participant only slightly decreased the variance explained by the model (from 34.5 to 28.1%, ΔAIC = 58.17), whereas excluding that for the unique FT in the stimuli decreased the variance explained fivefold (from 34.5 to 6.8%, ΔAIC = 776.1).

To interpret how SPIN and SNR affected the timing of FT-ITS, we completed *t* tests on the estimated marginal means (not corrected for multiple comparisons). For SPIN, low ability was associated with a 100-msec delay in the timing of FT-ITS as compared with high ability (*t*(14) = 2.2, *p* = 0.034). For SNR, the relationship with the timing of FT-ITS was U-shaped: FT-ITS were delayed in not only the hardest SNR condition (−4 dB) but also in the easiest SNR condition (8 dB). Specifically, in the −4-dB SNR condition, FT-ITS were delayed by 48 msec as compared with the 0-dB SNR condition (*t*(1426) = −2.2, *p* = 0.045), and by 39 msec as compared with the 4-dB SNR condition (*t*(1424) = −1.86, *p* = 0.064). In the 8-dB SNR condition, FT-ITS were delayed by 75 msec compared with the 0-dB SNR condition (*t*(1372) = −2.4, *p* = 0.015), and by 66 msec as compared with the 4-dB SNR condition (*t*(1369) = −2.2, *p* = 0.030). There was no difference between the −4- and 8-dB SNR conditions, nor between the 0- and 4-dB SNR conditions.

## DISCUSSION

In the present study, we undertook a preliminary exploration of whether the timing of FT-ITS is related to a HI individual’s perception of turn-taking cues in a conversation. Although mindful of the caveats that are discussed later, we found preliminary evidence that both the SNR of the conversation and the listener’s SPIN ability, two factors that should theoretically affect turn-taking perception, indeed had an effect on FT-ITS timing. To the best of our knowledge, this is the first indication of an influence of these factors on turn-taking perception and the first examination of this measure in a HI adult population.

As we predicted, people with lower SPIN ability showed delayed FT-ITS as compared with those with higher SPIN ability. Our prediction was based on previous research where FT-ITS timing was related to the clarity of turn-taking cues (Casillas & Frank 2012; Hirvenkari et al. 2013; Hidalgo et al. 2022). Our result thus extends the support for this relationship by implying a link between SPIN and turn-taking perception. That being said, this positive result comes with the caveat that we were unable to detect an effect of SPIN ability when this factor was implemented as a continuous individual predictor rather than with groups of high and low ability. This conflicting success underlines the need for replication of this result in future study, ideally with a larger sample size to mitigate the risk of insufficient statistical power.

Since the current investigation made use of a re-purposed dataset, we used the available SPIN data, but it may not have been ideal for our research question. Notably, the SPIN stimuli featured monosyllabic words with stationary noise, whereas our conversation stimuli contained multisyllabic, multiword utterances with a multitalker babble noise. With 10 talkers, this babble was relatively stationary but may have still contained some fluctuations in level. Dips in level can help with segregating speech from background noise—a strategy known as glimpsing (Cooke 2006). With conversation speech material, utterances follow a narrative and words are organized in a predictable way. Glimpsing may be especially beneficial here because it can provide listeners with sufficient speech information to build up contextual information and use this to fill in the speech material that they miss (Irsik et al. 2022). Considering this, we may have found a closer association between SPIN ability and the timing of FT-ITS if we had used sentences or continuous narrative material, instead of monosyllabic words, to test the former. Furthermore, a multitalker babble masker with few talkers, rather than stationary noise, would allow more opportunity for listeners to benefit from glimpsing, and thus potentially uncover more variation among participants in their ability to use this strategy.

Irrespective of differences in speech material and masker types, we would have expected SPIN ability and the timing of FT-ITS to be only moderately or weakly correlated. Although a SPIN measure typically only requires listeners to recognize words, our FT-ITS measure requires listeners to derive meaning from the speech, such that they can make predictions. We know from previous research that the ability to derive meaning is more strongly correlated with cognitive abilities, such as verbal information processing speed, reading comprehension speed, and working memory, than the ability to recognize words (Best et al. 2017). Building on this evidence, it may be that FT-ITS timing will be ideally predicted by a model that includes not only SPIN ability but that also incorporates cognitive abilities that are tied to conversation comprehension. This prediction is consistent with previous research, where FT-ITS timing was related to a listener’s ability to answer comprehension questions about a conversation (Hidalgo et al. 2022).

The effect of SNR on FT-ITS timing was non-monotonic: FT-ITS were delayed in conditions with both the highest (8 dB) and lowest (-4-dB) SNR, and no difference was detected between the two moderate (0- and 4-dB) SNR conditions. This was counter to our prediction that delayed FT-ITS would be associated with worse conversation perception. We expected participants to delay FT-ITS when turn-taking cues were obscured due to low SNR because: (1) It may take longer for the listener to accumulate sufficient evidence that the talker’s turn was ending, and (2) participants may have reduced cognitive resources for executing FT-ITS due to the increased perceptual load incurred by the degraded audio. These mechanisms are plausible explanations for the delay that we measured between the −4-dB SNR condition and each of the 0- and 4-dB SNR conditions. For the 0- and 4-dB SNR conditions, we speculate that we may have reached a ceiling improvement in the timing of FT-ITS. This suggests that the relationship between audio degradation and FT-ITS is not linear, but rather that there may be a threshold degree of audio degradation that must be reached to delay FT-ITS timing. A replication of this null effect in future research would help validate this interpretation.

Some caution is warranted with regard to the result associated with the 8-dB SNR condition, since this condition was the least tested, with only 4 of 17 participants providing data, contributing only 9% of the total data that was analyzed. Coincidentally, 3 of the 4 participants who completed this condition belonged to the high SPIN ability group (as can be seen in Table 1), which raises doubt about how the finding from this condition may generalize to a representative sample of hearing impairment. Nonetheless, the result for the 8-dB SNR condition presents a potentially interesting addendum to our original prediction that delayed FT-ITS would be associated with less clear turn-taking cues: This may only be the case when speech intelligibility is sufficiently challenged. One possibility may be that when speech is readily intelligible, the listener is no longer pressured to closely follow the turn-taking of the conversation because they do not need the visual cues to understand the speech. Given the limited sampling of the 8-dB SNR condition in the current dataset, directly testing this prediction here is impractical. The idea is consistent though with previous research that has shown that participants with NH look at the active talker in a conversation less often when speech is more intelligible (Hidalgo et al. 2022).

In any case, our pattern of results across all of the SNR conditions highlights that the timing of FT-ITS may only be sensitive to turn-taking perception within a specific range of audio degradation. This limited sensitivity would not be unusual—for example, it echoes the non-monotonic relationship that has been shown between pupil-dilation responses and changes in SNR during word recognition (Wendt et al. 2018). If this relationship proves to be true, it offers guidance for the design of future experiments on the timing of FT-ITS and turn-taking perception. Possibly, it may also help explain a recent null result, where FT-ITS timing was found to be similar between NH children and children with cochlear implants (Hidalgo et al. 2024). Following this interpretation, it could be that the FT-ITS timing of these two groups sampled behaviors at the two extremes of perception, missing the middle range where FT-ITS timing is (hypothetically) sensitive.

The current investigation applied an analysis procedure for classifying gaze data that was recently developed (Shiell et al. 2023). This procedure was created to be translated easily to future experiment setups that have low-precision and/or low-accuracy eye-tracking capabilities, such as a tablet device in an audiology clinic or classroom. It was ideal for the current dataset, where listeners’ heads were not fixed in space, and the absolute position of their gaze relative to the stimulus was not recorded. Although we lack a ground truth to validate this analysis approach, the gaze behaviors that it produced are in line with what has been observed in a previous study. With our criteria, we calculated that listeners looked to the active talker 59.6% of the time, which is within the range of what has been reported previously using a different experiment setup and analysis method: Specifically, Lu et al. (2021) reported that, while participating in a live interactive triadic conversation, HI listeners oriented their gaze toward the active talker between approximately 55 to 60% of the time (see Fig. 6 from Lu et al.), depending on the level of background noise (ranging from no background noise to 70 dB SPL).

As for the timing of FT-ITS, we observed that listeners executed FT-ITS with a median timing of 260 msec after the onset of the new talker’s turn, with 75% of the FT-ITS completed by 460 msec. This is substantially later than what was reported by Hidalgo et al. (2022), where the median FT-ITS of NH listeners occurred around the onset of the new talker’s turn (see Fig. 1 of that study). However, there the FTs were artificially set to have a consistent silent gap of 500 msec, leaving considerably more time for initiating saccades than in the current investigation, where the median silent gap duration was 280 msec. In another study, researchers looked at how the probability of gaze towards the new talker changed over time during FTs, rather than the timing of each FT-ITS. In NH listeners and without background noise, these studies report that this probability peaked at approximately 300 to 800 msec after the onset of the new talker’s speech (Tice & Henetz 2011; Casillas & Frank 2012; Foulsham & Sanderson 2013; Hirvenkari et al. 2013; Keitel et al. 2013; Dawson & Foulsham 2022). The FT-ITS timing reported in the current investigation, with 75% of the FT-ITS completed by 460 msec, falls within this range. That being said, it is difficult to make any conclusive comparison due to the substantial differences in methodology between these studies.

In the current investigation, we took an exploratory analysis approach but limited our hypothesis to focus on only one gaze behavior. Although this narrow focus was well-suited for our re-purposed dataset, we acknowledge that the behavior that we analyzed represents only one example of the variety of gaze behaviors that occur in response to dyadic conversation. Of note, the FT-ITS that we analyzed did not include the cases where listeners performed more than one ITS, no ITS, or ITS away from the new talker, which cumulatively occurred in 62% of the conversation FTs that we examined. This variety of behaviors attests to the complexity of real-world behavior and the potential for the discovery of other correlates of auditory perception. Building on our current hypothesis, one prediction may be that when turn-taking cues are less clear, listeners are less likely to make a FT-ITS, and more likely to engage in other gaze behaviors.

The current results provide preliminary support for our hypothesis that there may be a connection between turn-taking perception and FT-ITS timing in adults with HI, and thus represent a step towards the goal of using FT-ITS as a measure of the effect of hearing loss on real-life communication. This goal speaks to the need for outcome measurements with improved ecological validity in hearing science (Keidser et al. 2020). However, our measure of turn-taking perception misses an important element of real-world communication, namely the speech planning that occurs in parallel when a listener actively participates in a conversation, as is usually the case in everyday life. Unfortunately, our analysis method for predicting FT-ITS timing is not suitable for application with live conversation: As in our previous study (Shiell et al. 2023), we found here that predicting gaze behavior during FTs was greatly improved by the inclusion of each unique FT from the stimuli in the model via a random effects term. This random effects term essentially allowed the model to customize its fit for each unique FT in the stimuli, thereby controlling for variability in the data that is driven by stimuli variation. The role of this random effects term in the current model was substantial: When it was excluded from the model, the variance explained decreased from 34.5 to 6.8%, which highlights the relevance of stimulus-driven gaze variation in our dataset. This random effects term for unique FTs can only be modeled if each of the unique FTs are repeated to different participants, which is not possible when the conversation is live and the FTs are not repeatable. An important next step will therefore consist of examining the relationship between the timing of FT-ITS in response to prerecorded conversation, and measures of a person’s struggle participating in an interactive conversation under similar conditions. With such continued study, we hope that this research will advance our understanding of the real-world challenges experienced by HI listeners in their everyday lives.

## ACKNOWLEDGMENTS

The authors graciously acknowledge the work of Teresa Cabella and Simon With, who helped design the experiment and collected data in collaboration with M.M.S, and Soffi Skovlund, Josefine Hjort, and Caroline Esmann Busch, who provided audiological support and advice.
